# Stiffness-matched segmented metallic guidewire for interventional cardiovascular MRI

**DOI:** 10.1186/1532-429X-17-S1-P414

**Published:** 2015-02-03

**Authors:** Burcu Basar, Adrienne E Campbell-Washburn, Toby Rogers, Merdim Sonmez, Anthony Z Faranesh, Kanishka Ratnayaka, Robert J Lederman, Ozgur Kocaturk

**Affiliations:** 1 Division of Intramural Research, National Heart, Lung and Blood Institute, National Institutes of Health, Bethesda, MD, USA; 2 Institute of Biomedical Engineering, Bogazici University, Istanbul, Turkey

## Background

Conductive guidewires and intravascular catheters are at risk of RF-induced heating under MRI [1]. Heating is found predominantly at the tip of conductive wires [2], and is modulated by wire diameter, length and insulation thickness [3]. Non-conductive materials, such as polymer, impart unsatisfactory mechanical properties on guidewires in terms of flexibility, stiffness, and torquability, for navigating tortuous cardiovascular structures and for safely delivering catheter devices.

We developed a novel MRI guidewire design that avoids RF heating yet preserves the mechanical features of conventional X-ray guidewires. Short non-resonant segments of nitinol are connected using stiffness-matched insulated notched couplers, preventing standing wave formation yet appearing mechanically indistinguishable from nitinol guidewires.

## Methods

**Figure 1:**
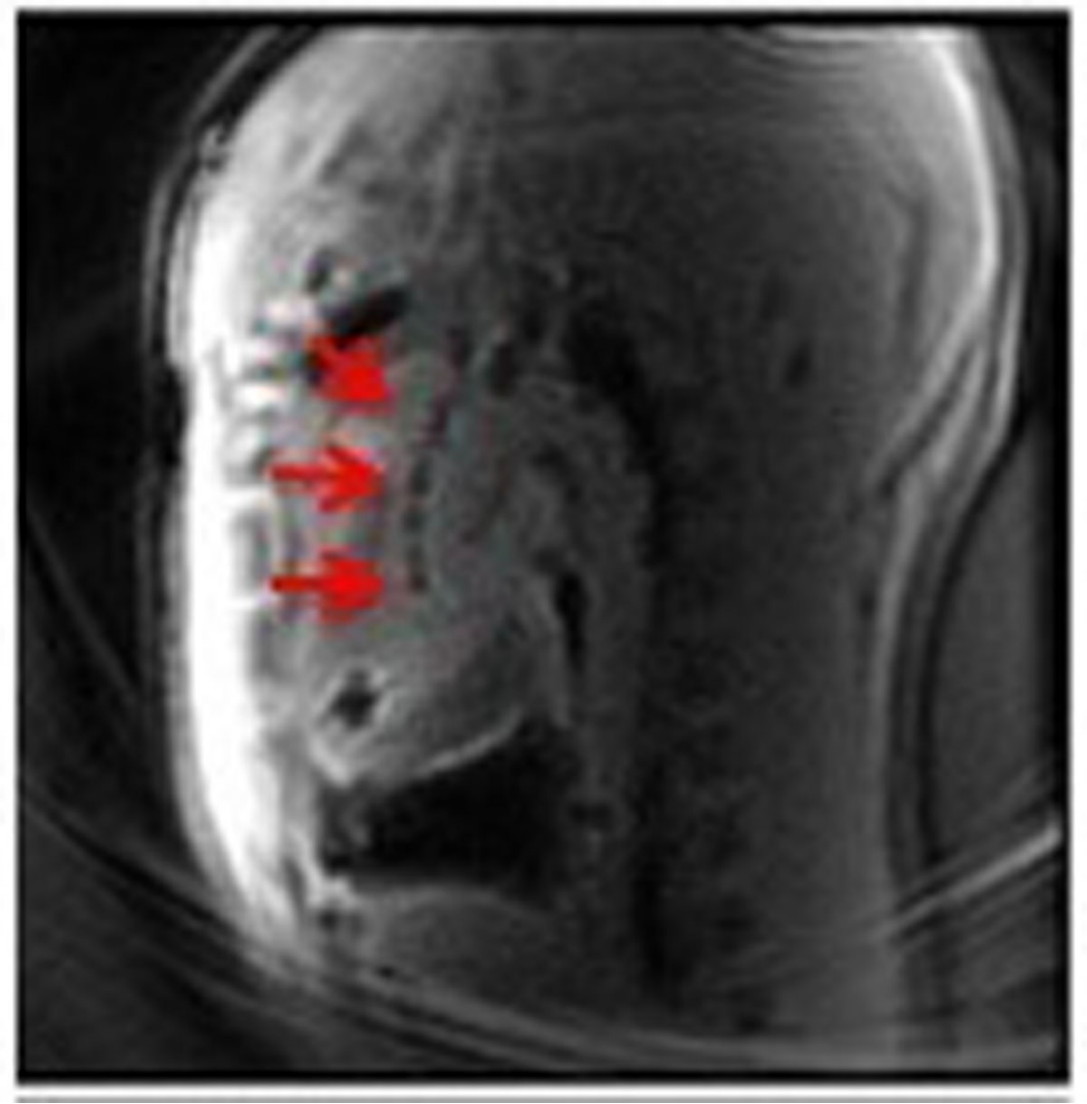
**In vivo left heart catheterization in swine using MRI safe guidewire design.** Iron-oxide markers on device tip shown with red arrows. TE/TR=1.9/4.2 ms, slice thickness=6mm, matrix=192x192, flip angle=15

The core of a passive 120cm guidewire was constructed from nitinol rod segments shorter than a quarter-wavelength in vivo at 1.5T (10cm). The segments were joined by electrically insulated nitinol tubes. A surrounding outer braided polymer enhanced torque response, insulation and safety. The distal tip was insulated using non-braided polymer and low durometer thermoplastic polymer for flexibility and trackability. Iron-oxide markers created MRI susceptibility artifacts for enhanced visualization *in vivo* in a swine (Fig 1).

RF heating was measured in an ASTM F2182 gel phantom. Tip and shaft temperature was measured using a fiber optic probe (*OpSense*) at 1.5T (*Aera*, Siemens) [4]. Heating was measured at high flip angle (75°) bSSFP (TR/TE, 2.88/1.44 ms; thickness, 6 mm; FOV, 350×350 mm; matrix, 192×144) and compared to a custom non-segmented nitinol core wire with identical jacketing serving as a control.

## Results

**Figure 2:**
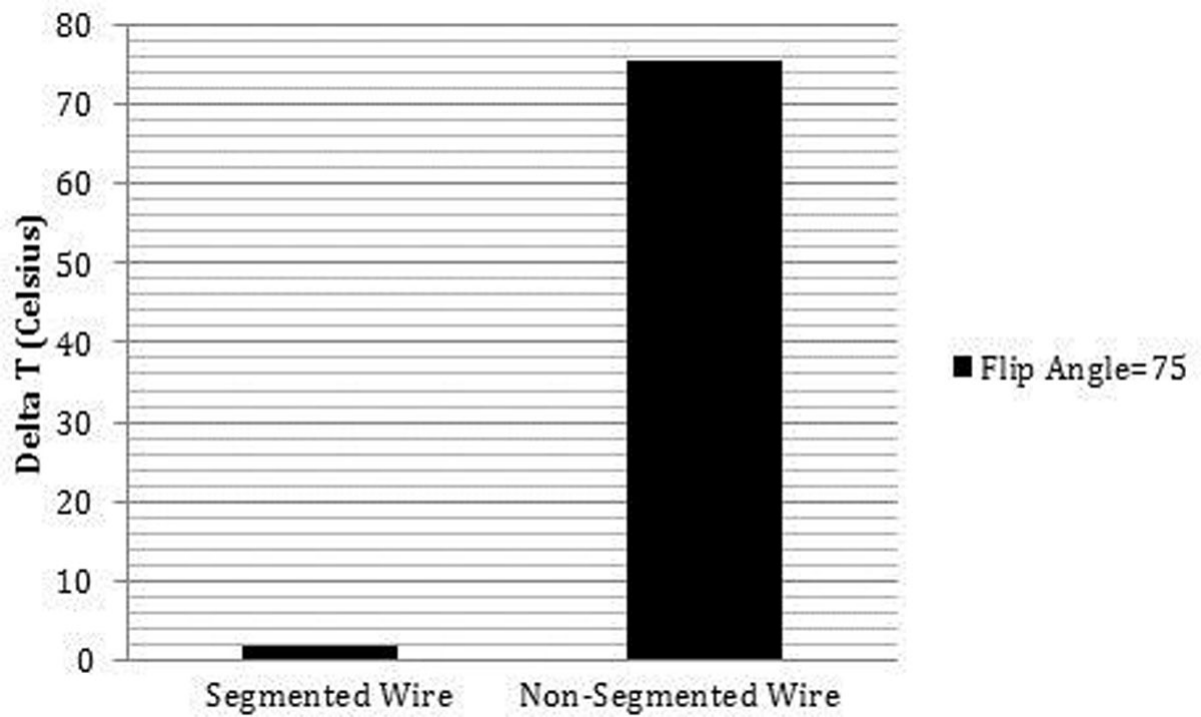
Deviation from baseline temperature readings acquired over 30 seconds prior to scanning during *in-vitro* heating experiments.

The segmented MRI guidewire exhibited a maximum temperature increase of 1.6°C at the tip, compared with 74°C for the non-segmented comparator, during a 60s scan at a flip angle of 75° (Fig 2). Systematic temperature measurements along the shaft detected negligible heating, confirming successful electrical insulation by the inter-segment connectors. Trackability in a tortuous vascular phantom resembled commercial comparators (*Glidewire*, Terumo).

## Conclusions

We demonstrate a simple and intrinsically safe new design for passive metallic MRI guidewires. The guidewire exhibits negligible heating at high flip angles in conformance with ISO standards (<2°C) [5], yet mechanically resembles a high-performance conventional metallic guidewire. This may represent a significant advance once applied in clinical MRI catheterization.

## Funding

Supported by NHLBI Z01-HL006041. BB, OK, and RJL are co-inventors on patent applications assigned to NIH.
